# Brain asymmetry in the white matter making and globularity

**DOI:** 10.3389/fpsyg.2015.01355

**Published:** 2015-09-10

**Authors:** Constantina Theofanopoulou

**Affiliations:** Department of General Linguistics, Universitat de Barcelona, BarcelonaSpain

**Keywords:** brain asymmetry, lateralization, skull, globularity, corpus callosum, white matter, brain rhythms, language

## Abstract

Recent studies from the field of language genetics and evolutionary anthropology have put forward the hypothesis that the emergence of our species-specific brain is to be understood not in terms of size, but in light of developmental changes that gave rise to a more globular braincase configuration after the split from Neanderthals-Denisovans. On the grounds that (i) white matter myelination is delayed relative to other brain structures and, in humans, is protracted compared with other primates and that (ii) neural connectivity is linked genetically to our brain/skull morphology and language-ready brain, I argue that one significant evolutionary change in *Homo sapiens’* lineage is the interhemispheric connectivity mediated by the Corpus Callosum. The size, myelination and fiber caliber of the Corpus Callosum present an anterior-to-posterior increase, in a way that inter-hemispheric connectivity is more prominent in the sensory motor areas, whereas “high- order” areas are more intra-hemispherically connected. Building on evidence from language-processing studies that account for this asymmetry (‘lateralization’) in terms of brain rhythms, I present an evo-devo hypothesis according to which the myelination of the Corpus Callosum, Brain Asymmetry, and Globularity are conjectured to make up the angles of a co-evolutionary triangle that gave rise to our language-ready brain.

## Introduction

**FIGURE 1:**
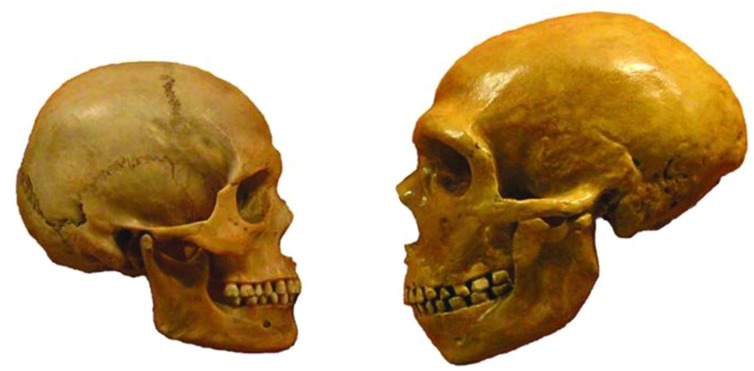
**Illustration of the hypothesis: observable skull differences between anatomically modern human **(left)** and Neanderthal (right)**.

The general aim of this paper is to support the idea that the key underlying our human- specific cognitive profile is to be found in the changes that brought about a more globular brain shape. As far as I can tell, two scientific hypotheses have already been put forward claiming that it was essentially this globular shape that determined the brain profile of *Homo sapiens*: one, by Boeckx and Benítez-Burraco, 2014a,b), called it “*globularity*” and hypothesized that modifications in the fronto-parieto-thalamic network ought to be taken into account; the other, by Hublin et al. (2015), called it the “*globularization phase*,” focusing, thus, on the developmental phase of the shaping, which, according to their findings, is due to bulging parietal and occipital bones (see **Figure 1**).

These two hypotheses can be understood as viewing the same changes from different perspectives. While Hublin et al. (2015) concentrate mostly on the evolutionary (occipito-parietal protrusion) and developmental (cerebellum) facet of the problem, Boeckx and Benítez-Burraco, 2014a,b; Benítez-Burraco and Boeckx, 2015) mainly focus on the link between globularity and language on a genetic level: their positing that the fronto-parieto-thalamic network might be of relevance seems to reflect the great majority of the findings that implicate the fronto (B44/45)-parietal (BA22) lobes in linguistic processing, broadening this cortical network to its subcortical “afferent and efferent expansion,” namely the thalamus (Buzsáki, 2006; Theofanopoulou and Boeckx, 2015). The integration of the thalamus should not strike us as irrelevant at all, as it essentially relays the cerebellar input to the frontal lobe (BA44/45 included; for cerebello-thalamic connectivity: Leiner et al., 1989; Schmahmann, 1997; Engelborghs et al., 1998, for thalamico-BA44/45 connectivity: Ford et al., 2013; Bohsali et al., 2015) and its growth is correlated to associated expanded areas, like the parietal lobe (and more specifically the novel precuneus, Cavanna and Trimble, 2006; Bruner, 2014; Bruner et al., 2014). By the logic of co-evolution (namely that two tightly connected brain parts exert pressure to each other, affecting each other’s evolution), it can be deduced that both the cerebellum and the thalamus are crucially involved in the cognitive mechanisms that result in language.

In this paper, however, I will take the expansion of the cerebellum to be the guiding line for the following reasons: firstly, the cerebellum is directly connected to the evolutionarily- significantly splayed parieto-occipital bone; secondly, it is particularly in these posterior sensorimotor regions of the cortex where the Corpus Callosum permits interhemispheric connectivity thanks to its anterior-to-posterior increase in size, myelination and fiber caliber (Aboitiz et al., 1992; Doron and Gazzaniga, 2008); thirdly, this connectivity is taken to be crucial for permitting the rhythmic interhemispheric interplay observed in language processing sensorimotor networks (Morillon et al., 2010).

The frontal cortex will not figure much in my core hypothesis, as according to Barton and Venditti (2014), even though absolute and proportional frontal region size increased rapidly in humans, this change was tightly correlated with corresponding size increases in other areas and overall brain size; besides, research has demonstrated that the parieto-occipital fossa’s protrusion was the most decisive; lastly, as regards to evolutionary changes in frontal connectivity, Neubert et al. (2014) have shown that actually what differentiates humans’ and macaques’ frontal cortex is its coupling to posterior auditory areas (which is much stronger in humans), something that seems to justify my attention toward the posterior sensorimotor areas.

**FIGURE 2:**
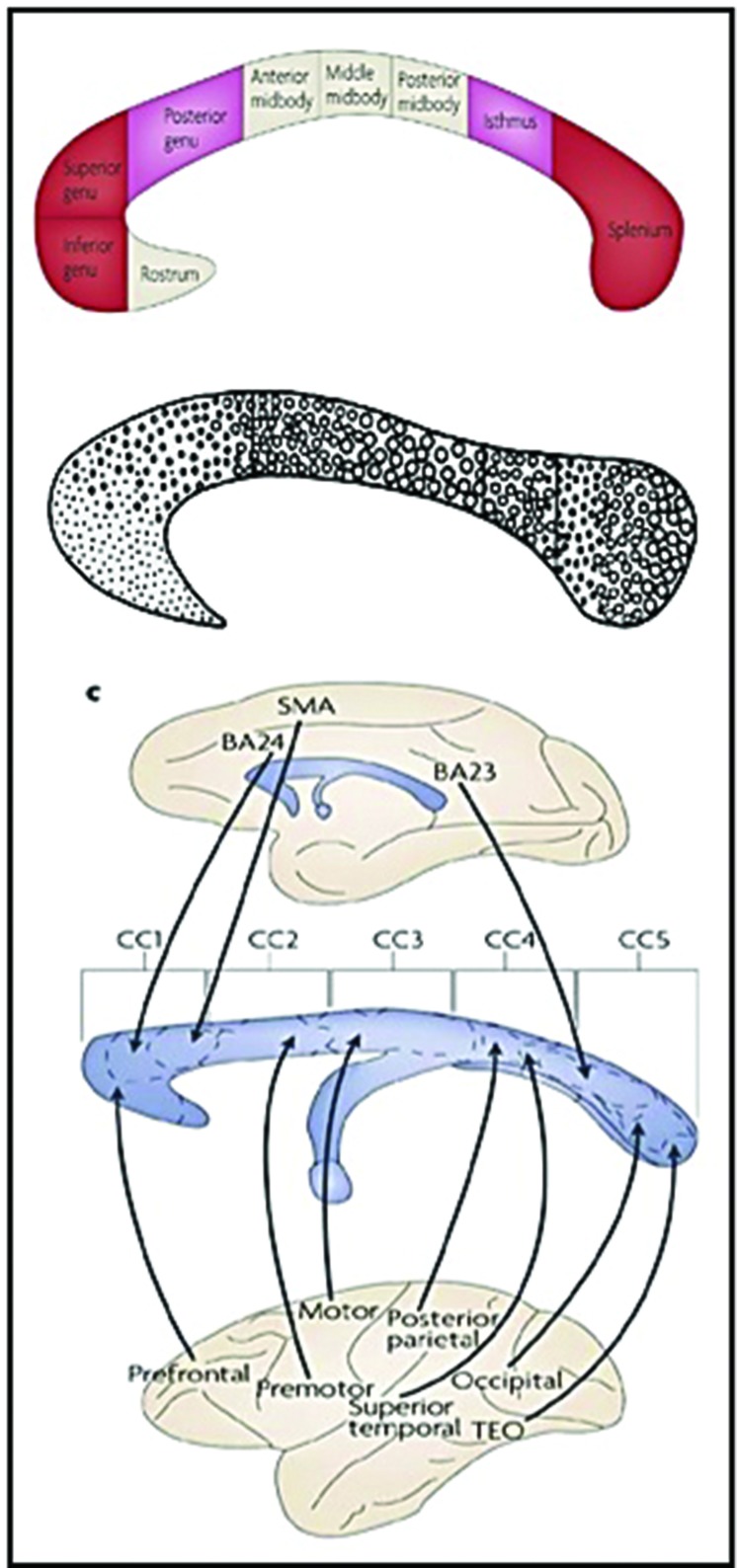
**The structure of the Corpus Callosum permits interhemispheric connectivity in the posterior cortex.** (Composition of pictures modified from: Aboitiz et al., 2003; Paul et al., 2007; Uhlhaas and Singer, 2010b.)

Ultimately, my objective is to bring out how -under this novel perspective- we could also make sense of the intricate idea of Brain Asymmetry (‘Lateralization’) and, thus, elucidate the hitherto unexplored relation of Brain Asymmetry-Corpus Callosum-Globularity. More specifically, I will argue that the architecture of the Corpus Callosum, allowing for interhemispheric connectivity in the posterior cortex (posterior temporal- posterior parietal- occipital cortex) and for intrahemispheric connectivity in the anterior- medial cortex (frontal- anterior parietal cortex-anterior/medial temporal cortex), suggests a novel way of capturing how brain asymmetry (a phylogenetically common trait) made it possible for our language- ready brain to arise (see **Figure 2**).

The structure of the paper will be the following: first, I will briefly introduce the idea of Brain Asymmetry- Lateralization (see Brain Asymmetry – Lateralization) and explain how it fits in the framework I wish to put forward; then I will look into how Asymmetry can be captured in terms of brain rhythms and analyze how the morphology of the Corpus Callosum renders this asymmetry possible (1.1). Next, I will draw attention to alpha and beta rhythms and propose a way in which they could constitute an overlooked window into both Ontogeny and Phylogeny (1.2), adducing supporting data from deficits that could be seen as speech-related oscillopathies (1.3). In Section “White Matter- Globular Brain Pattern,” I will delve into the idea of the White Matter begetting our Globular Brain Pattern during development. In the end, I will discuss how the posterior brain and skull co-evolved in *H. sapiens* (see Posterior Brain and Skull Enlargement in *Homo sapiens*).

### Brain Asymmetry – Lateralization

Brain Asymmetry, long thought to be human- specific, has been shown to lie along an evolutionary continuum (Fitch and Braccini, 2013), so that even the first biological pillar of the uniqueness of human language, namely its strong left-lateralization (Lenneberg, 1966) has fallen down. Comparative studies have suggested a left-hemispheric dominance for conspecific communication in a wide variety of species (Ocklenburg and Güntürkün, 2012), such as chimpanzees (Taglialatela et al., 2008), rhesus monkeys (Hauser and Andersson, 1994), dogs (Siniscalchi et al., 2008), mice (Ehret, 1987), sea lions (Böye et al., 2005), and frogs (Bauer, 1993). More tellingly, a left-dominance has been reported in canaries as regards to hypoglossal functions (Nottebohm, 1971), in zebra finches concerning vocal learning (Voss et al., 2007; Moorman et al., 2012) and in Bengalese finches for song discrimination (Okanoya et al., 2001). Another shared dominance worth mentioning is that of emotional processing in the right hemisphere (e.g., Önal-Hartmann et al., 2011). There is evidence that it obtains also in gelada babboons (Casperd and Dunbar, 1996), mangabeys (Baraud et al., 2009), rhesus macaques (Vermeire and Hamilton, 1998), chimpanzees (Parr and Hopkins, 2000), marmosets (Hook-Costigan and Rogers, 1998), and dogs (Siniscalchi et al., 2008).

Last but not least, asymmetry in motor behavior and more concretely, in handedness, has been erroneously thought to be human- specific and furthermore to imply -along with language- a general left hemispheric dominance common to humans (Harris, 1991). On the one hand, left-motor-lateralization is not unique to humans: as Smaers et al. (2013) review, lateralization in motor behavior has been found in primates (Nudo et al., 1992; Bogart et al., 2012; Sun et al., 2012), non-primate mammals (Ehret, 1987; Rogers et al., 1994), birds (Vallortigara and Andrew, 1994), fish (Cantalupo et al., 1995; Bisazza et al., 1997), reptiles (Engbretson et al., 1981; Hoso et al., 2007) and amphibians (Bauer, 1993; e.g., pawedness in toads Bisazza et al., 1996), footedness in birds (Rogers and Workman, 1993) and finnedness in fish (Hori, 1993). Handedness, in particular, was recently shown to be present also in non-primate mammals (bipedal marsupials), something that challenges the notion that ‘true’ handedness is unique to primates (Giljov et al., 2015). On the other hand, the right- handedness rule would imply “*that most left handed people display right hemispheric dominance for language, an assertion not validated by rigorous empirical studies* (Knecht et al., 2000),” as Washington and Tillinghast (2015) observe. Ocklenburg and Güntürkün (2012) suggest there are both genetic and epigenetic factors we should take into account in the context of brain asymmetry. They provide the example of pigeons: their determined embryonic egg- position (genetic factor) permits only their right eye to be stimulated by light (epigenetic factor), resulting in left hemisphere superiority for visual object discrimination. (For a good experiment on how early navigational experience in pigeons affects lateralization, see Mehlhorn et al., 2010.) The authors finally suggest that similar “*early spinal asymmetries could act as lateralized “precursors” of asymmetrical cortical motor functions*” in humans (such as prenatal bias on turning the head to the right); epigenetic factors though should not be overlooked, as their relevance in human handedness is much more important (unlike birds in the case of vision, humans are not genetically confined to using only one hand!). The lower incidence of left-handedness in countries where the left hand is associated with uncleanliness is a good example portraying how much epigenetic factors affect handedness (Zverev, 2006). Siding with Benítez-Burraco and Longa (2012), I conclude that “*the relationships between right-handedness (structural and functional) brain lateralization, and language are perhaps not significant enough, or illuminating from an evolutionary perspective.*”

With the aforementioned I wish to underline that human language lateralization is not due to a dominance of the left hemisphere for language as such: none of the hitherto known cognitive functions emerged during hominin evolution. Rather, they are phylogenetically shared, as one should expect given the conservation of brain rhythms across a wide range of species (Buzsáki et al., 2013; Boeckx and Theofanopoulou, 2015). This should lead us to consider the following: given that gray matter-subcortical parts of the brain are associated to sensorimotor and cognitive functions, and white matter modulates the distribution of action potentials among them and the neocortex (Fields, 2005, 2008), it is probably this modulatory function that gives to the core-cognitive functions (gray matter) the level of complexity detected only in our species and required for language.

I agree with Ocklenburg and Güntürkün (2012) in that white matter might be an overlooked window to brain asymmetry- issues: “*A common conception is that functional asymmetries are a consequence of structural asymmetries in the brain ……research…has focused on macroscopic gray matter asymmetries… evidence from recent studies in animal models suggests that structural asymmetries in connectivity patterns of homologous regions in the two hemispheres may be of greater functional relevance.*” Another reason to believe so is the developmental nature of white matter’s myelination; occurring relatively slowly over the lifespan (Hynd et al., 1995), white matter constitutes a perfect mirror candidate of the developmental nature of human language acquisition. Besides, as I noted above, the chemical mechanisms of myelination are decisive for axons’ generating action potentials, something that can be directly associated with the oscillatory basis of language I will shortly highlight.

**FIGURE 3:**
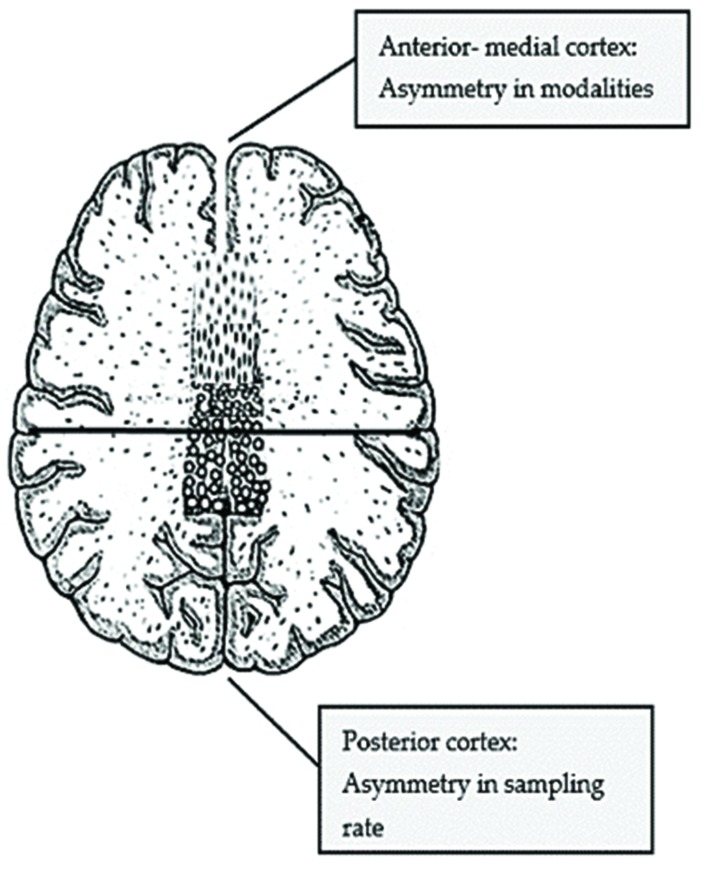
**Illustration of the Brain Asymmetry Hypothesis (made by the author)**.

In what follows, I will try to illustrate the relevance of the brain’s largest white tissue structure, i.e., the Corpus Callosum, in Brain Asymmetry, and afterward provide a link to Globularity. Let me clarify that the Corpus Callosum was not chosen merely because of its size, but because of its decisive position in the brain and its human- specific structure. The size, myelination and fiber caliber of the Corpus Callosum presents an anterior-to-posterior increase (Doron and Gazzaniga, 2008), resulting in interhemispheric connectivity being more prominent in the sensory motor areas, whereas “high- order” areas are more intrahemispherically connected (**Figure 3**). Studies comparing humans’ and monkeys’ corpora callosa tellingly revealed that in humans the proportion of large diameter fibers in callosal regions that interconnect primary sensory areas is higher than in macaques (Aboitiz et al., 1992) and that the fiber organization has nothing in common with the callosal organization reported in monkeys (Jones et al., 1978; Killackey et al., 1983).

The hypothesis I wish to put forward is the following: it is the structure of the Corpus Callosum that makes humans’ brains display a sophisticated, selective asymmetry: in the anterior/medial cortex, where callosal fibers are narrow and intrahemispheric connectivity is enhanced, asymmetry is expressed at the level of small- world networks, i.e., cognitive functions appear to be lateralized as modules (e.g., the default network in the left and the attentional in the right hemisphere: Wang et al., 2008; De Schotten et al., 2011); in the posterior cortex, there is no such asymmetry, as visual, auditory, and motor functions appear in both hemispheres. What makes the posterior cortex asymmetrical is to be found in the hemispheres’ refinement toward processing input of specific ‘sampling rate’ (temporal-faster rate sampling executed in the left hemisphere and spectral-slower rate in the right). In the coming section, data fostering my hypothesis will be adduced (see **Figure 3**).

#### Asymmetry in the Dynome and the Corpus Callosum

Contemporary neural models of auditory language processing proposed that the two hemispheres are differently specialized in either temporal (left hemisphere) or spectral (right hemisphere) resolution (Zatorre et al., 2002), or in other terms that they differ in terms of their preferred “sampling rate” with the left hemisphere being well suited for faster- rate sampling and the right for slower rate (Poeppel, 2003; Hickok and Poeppel, 2007, 2015; Morillon et al., 2010). According to Celesia and Hickok (2015), “*These two proposals are not incompatible as there is a relation between sampling rate and spectral vs. temporal resolution: rapid sampling allows the system to detect changes that occur over short timescales, but sacrifices spectral resolution, and vice versa.*”

**FIGURE 4:**
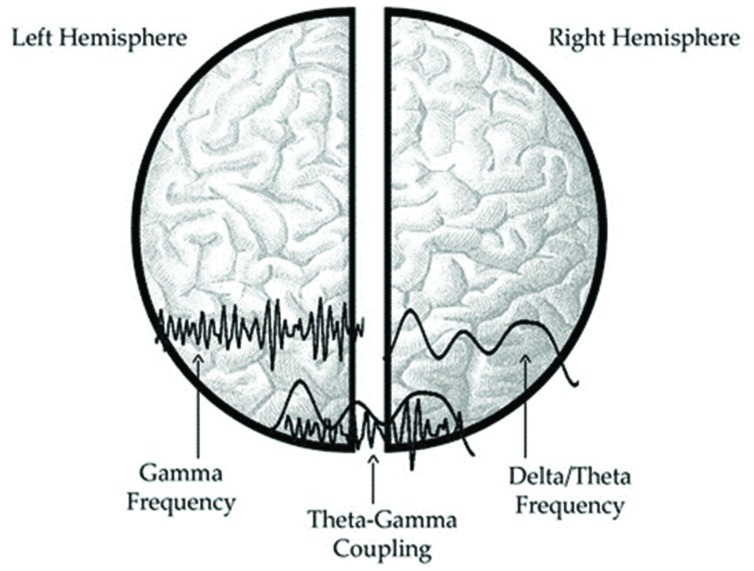
**Illustration of the AST hypothesis (Theofanopoulou Mikaella; Rights reserved)**.

More concretely, Morillon et al. (2010) found that there are two auditory speech sampling mechanisms working in parallel: while syllabic parsing of the input (slow- rate delta-theta oscillations, ~4 Hz) is predominantly assigned to the right hemisphere, the left hemisphere has been shown to have a primacy for the processing of phonemic input (fast- rate gamma oscillations, ~40 Hz; see **Figure 4**). A dynamic interplay is assumed to allow for the timely coordination of both information types, namely for fast phonemic gamma being modulated by syllabic theta oscillations. The hypothesis of functional asymmetry concerning hemispheres’ preferential cues is known as the AST (asymmetric sampling in time) hypothesis (Poeppel, 2003; Ghazanfar and Poeppel, 2014).

Tellingly, even though this asymmetry was most pronounced in the auditory cortex, motor areas also express natural oscillatory activity that corresponds to the same rates: intrinsic jaw movements oscillate at delta/theta oscillations, thus presenting an overlapping parsing with the syllabic network, while the phonemic fast gamma oscillations underlie tongue and formant transition movements (e.g., trill at 35–40 Hz; Morillon et al., 2010).

Another significant finding of the experiment conducted by Morillon et al. (2010) was a strong intrinsic asymmetry (also manifest at rest) between the articulatory (left hemisphere) and the hand motor cortex (right hemisphere). This asymmetry is suggested to be phylogenetically “inherited,” probably because of the long shared sinistral pharyngeal muscle control on the one hand and the dextral hand gestures control on the other. (Let me parenthesize here to point out from another perspective that erroneously right- handedness has attracted all the attention: what is significant for language is hand gestures accompanying language and not handedness *per se.*)

In addition, there are cases of other, non-human, even non-vocal-learning species, whose lip- smacking is tuned into the same slow oscillatory cycles, present in human syllabic sampling (such as the Gelada Baboons *Theropithecus gelada*, Bergman, 2013, see also Ghazanfar et al., 2012). It would then be critical to find out whether the Gelada Baboons display also a human- like right dominance of these lip movements and whether they are coupled with other faster oscillatory cycles, subserving communicatory processes. With the latter, I don’t mean to imply that our linguistic profile is due to our capacity of housing spectral and temporal information within a narrow time-window, since this competence is again found to be present in other species: mustached bats exhibit the same oscillatory asymmetry in echolocation processing (Washington and Tillinghast, 2015). Rather, what all these phylogenetic observations are meant to highlight is that we indeed share both generic and elemental mechanisms with other species: the key to our questions is to be found in how *H. sapiens* ‘coupled’ the modalities inherited. Adopting a Darwinian thinking, it seems indeed plausible that the connectivity across and within modalities afforded by the peculiar structure of the Corpus Callosum is evolutionarily significant. In the case of auditory processing, it has been experimentally shown that it is the posterior Corpus Callosum that gives rise to this linguistically- crucial theta- gamma coupling (Rumsey et al., 1996; Pollmann et al., 2002; Nosarti et al., 2004). Even early dichotic listening experiments on patients with Corpus Callosum abnormalities had specified that agenesis in the splenium is pertinent to aberrant auditory interplay (Sugishita et al., 1995; Pollmann et al., 2002). Friederici et al. (2007) tellingly observe that “*an intact posterior third of the C[orpus] C[allosum] connecting temporal regions is a necessary precondition for a prosody-induced N400 mismatch effect. Lesions in the anterior two-thirds of the CC that connect frontal regions, in contrast, can cause a modulation of the prosody- induced mismatch effect but cannot eliminate the effect.*” Sammler et al. (2010) conducted an experiment with two groups of patients with lesions either in the anterior or the posterior Corpus Callosum: the latter did not exhibit the expected mismatch between segmental (temporal) and suprasegmental (spectral) features of language.

Apart from the role of the Corpus Callosum in auditory processing, all these studies also hint at the involvement of the right hemisphere in language processing (for a review see Lindell, 2006). Fully consistent with my hypothesis, Overath et al. (2015), after finding that the effect of speech segment length was robust in both hemispheres, inferred the following: “*It is therefore possible that laterality effects are driven more by higher order linguistic processing demands than by speech analysis per se.*”

**FIGURE 5:**
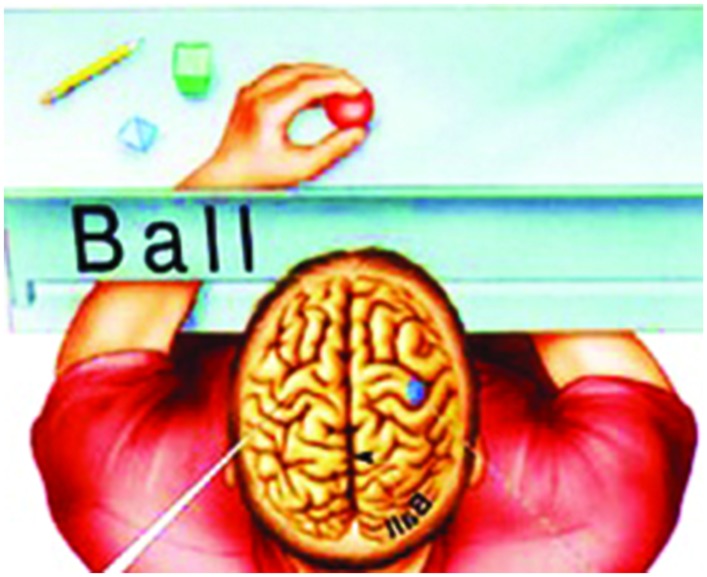
**A split- brain patient demonstrates speech auditory comprehension (‘Ball’), by picking the ball with his left hand (right motor control; Adapted from: http://thebrain.mcgill.ca/flash/capsules/experiencebleu06.html)**.

From this perspective, we can also explain the initial results from split- brain patients (Corballis, 1998), that apparently confirmed the linguistic incompetence of the right hemisphere: callostomy patients could not verbally answer to language questions presented to isolated right hemispheres, because the articulatory (only) ability is left- dominant. However, when asked for non-verbal responses, the patients demonstrated speech auditory comprehension, by picking with their left hand (right motor control) the object (uttered by the experimenter) among an array of objects (see **Figure 5**).

Lastly, the idea of lateralization in terms of synchronic activity in the posterior cortex -mediated by large- diameter, fast- conducting callosal regions- was firstly formulated by Aboitiz et al. (2003). However, back then there was no evidence reporting a right- dominance for spectral and a left- dominance for temporal processing, so that the authors made the following conclusion: “*There is not yet evidence for the existence of synchronic ensembles during performance in working memory tasks, but 40 Hz synchronic oscillations have been reported during linguistic performance… The role of synchronic activity in working memory processes, be they linguistic, auditory or visual, urgently needs to be investigated.”* The proposal of dynamic asymmetry presented here should be really close to what Aboitiz et al. (2003) had in mind.

#### Alpha and Beta Rhythms- a Window to Ontogeny and Phylogeny of Mirror Neurons

Although research has mostly focused on the rhythms that have a discernible effect in auditory processing (delta/theta and gamma oscillations), alpha and beta rhythms are suggested to be equally implicated but in a reverse mode: by being suppressed.

This shift of interest toward the significance of these two rhythms is apparent if one pays attention to the way alpha/beta suppression is treated by Poeppel and colleagues: Doelling et al. (2014) investigated to what extent the oscillation-based envelope tracking (discussed above) also mediates the relationship between sharpness and intelligibility; the results supported this correlation but the authors also noted that another rhythm (alpha) was suppressed in their experiment. They limited their observation to mentioning Obleser and Weisz (2012), who have shown that alpha power suppression is related to intelligibility, but 1 year after they sought to put this to test (Luc and Arnal, 2014). Interestingly, they found that the typical increase in theta band was always followed by a broadband suppression of alpha (9–14 Hz) and beta (15–25 Hz) bands and suggested that “*the brain exploits the level of post-stimulus alpha suppression as internal evidence to determine how well the stimulus matched the prediction.*”

If we now turn back to the experiments made by Obleser and Weisz (2012), alpha rhythm seems to have far-reaching implications in auditory processing: on the one hand, it has been shown to be suppressed to reinforce acoustic intelligibility, but on the other hand, alpha rhythm is enhanced during auditory memory retention (Knecht et al., 2000; Jensen et al., 2002; Obleser et al., 2012; Theofanopoulou and Boeckx, 2015). These findings suggest that alpha rhythm moderates the interplay between working memory/attention and intelligibility. Tellingly, when the auditory memory is overloaded (hence alpha rhythm enhancement is further employed), acoustic degradation affects processing, because of the non-canonical ellipsis of alpha suppression (Obleser et al., 2012). This is also consistent with studies reporting an increased activation of the cerebellum in high- load tasks, suggesting a prominent role of the cerebellum in working memory processing (Kirschen et al., 2010; Stoodley et al., 2012; Luis et al., 2015).

On the grounds that Morillon et al. (2010) regard the synchronization of the motor (jaw movements/trill or formant transitions) and auditory (syllabic/phonemic) modalities as significant, I take it that there must be such a correlation also between the motor and auditory alpha and beta suppression. This conclusion can be reached thanks to studies focusing on the Mirror Neurons System: a system of neurons which were first thought to be activated only during action execution and action-observation (Rizzolatti and Craighero, 2004). However, recent experiments leave no doubt that mirror neurons (MN) are also implicated in auditory processing (Cuellar et al., 2012) and sensorimotor learning (Catmur et al., 2007; Hickok and Hauser, 2010). At present, it is acknowledged that MN integrate cross- modal information and crucially all the information that has been said to be involved in language processing (Senkfor, 2002; Molnar-Szakacs and Overy, 2006).

What is even more relevant to the present paper is that EEG and MEG studies report a suppression of alpha/mu and beta- band activity in the sensorimotor area, among other areas (Rizzolatti and Luppino, 2001; Muthukumaraswamy and Johnson, 2004; Ulloa and Pineda, 2007; Pineda, 2008; Perry and Bentin, 2009; Perry et al., 2010; Frenkel-Toledo et al., 2013; Lange et al., 2015; from now on, I will focus on the mu-alpha rhythm suppression, as beta- band suppression has only very recently been shown to be involved in the context of MN; see Lange et al., 2015).

There is corroborating evidence that alpha/mu rhythm desynchronization in the sensorimotor system appears early in infancy and its functional properties are so strongly modulated by maturation, that the sensorimotor system evolves from a random (in infants) to a “small- world” organization (in children and adults; Ferrari et al., 2009; Marshall and Meltzoff, 2011; Cuevas et al., 2014; Berchicci et al., 2015). This “small- world” networking is achieved by a wiring pattern in the brain that is thickly intra-connected locally and sparsely interconnected globally (Changizi, 2001, p. 571; Karbowski, 2003; Sporns and Kötter, 2004; Sporns and Zwi, 2004). Pineda (2005) proposed that “*mu rhythms represent an important information processing function that links perception and action-specifically, the transformation of ‘seeing’ and ‘hearing’ into ‘doing’.*”

In addition, if we go back to Morillon et al. (2010), they suggest that “*inherent auditory- motor tuning at the syllabic rate and acquired tuning at the phonemic rate are also compatible with two recognized stages of language development in infants; an early stage with production of syllables that does not depend on hearing (also observed in deaf babies), followed by a later stage in which infants match their phonemic production to what they hear in caregiver speech.*” It seems to me that alpha/mu rhythm’s maturation in development could indeed be the key for the interplay of the two sampling rates discussed, given that it is directly connected to the maturation and myelination of the white matter: Jann et al. (2012) found positive correlations of Fractional Anisotropy with alpha frequency within the splenium of the corpus callosum. Let me reiterate that the splenium, being at the posterior-myelinated part of the Corpus Callosum, presents an overlap of myelin water fraction with Fractional Anisotropy values within their thick axons that permits fast signal conductance. It is, furthermore, noteworthy that Miller (1994) sees such a connection between myelination, alpha frequency and intelligence that he put forward a brain myelination hypothesis of intelligence.

More importantly, these ontogenetic observations can be linked also to phylogenetic issues, which could result in interesting future experiments. It is known that MN were originally detected in monkeys’ area F5. What is not yet appreciated is that MN respond to the observation of lip- smacking and hand- actions (Ferrari et al., 2003) by inhibiting mu rhythm (Vanderwert et al., 2013; Cook et al., 2014). Given that there are hypotheses -in the context of human ontogeny- proposing that MNS in humans pass from being purely visual to multimodal (Iacoboni and Dapretto, 2006; Ferrari et al., 2009), it could be conjectured that this developmental shift, mediated by the myelinated posterior Corpus Callosum, was crucial for our linguistic cognition. (For data fostering this idea, see Autism Spectrum Disorder.)

#### Deficits as Speech- Related Oscillopathies

##### Autism spectrum disorder

It shouldn’t strike us as strange that a plethora of evidence in line with the above comes from autism, where actually the developmental process is most obviously affected. Murphy et al. (2014) found that alpha- band deployment was severely impaired, giving rise to increased distraction, and Jochaut et al. (2015) encountered that ASD patients, instead of down- regulating gamma activity by theta, presented an opposite dependency such that gamma and theta- coupling jointly increased out of physiological ranges.

In light of what has been said about the implication of these rhythms in speech processing, it is clear that dysfunctional theta/gamma coordination and alpha suppression would disrupt the alignment of neuronal excitability with syllabic onset, compromising speech decoding.

The data seem also in consonance with what has been conjectured before about an earlier visual system which later becomes multi- modal: Damarla et al. (2010) show that ASD patients display more activation in visuospatial (bilateral superior parietal extending to inferior parietal and right occipital) areas, something that possibly indicates a compensatory role of visual processing during speech perception. The latter is supported by experiments according to which ASD subjects extensively explore the mouth region in face- to-face situations (Klin et al., 2002), and use specific attention modes to enhanced local visual processing (Schwarzkopf et al., 2014).

Regarding the Corpus Callosum in ASD, there is a aboundance of studies proving that its size is degenerated in the posterior areas (Alexander et al., 2007; Just et al., 2007, among others). Moreover, the fact that abnormalities in the parietal lobes (Courchesne et al., 1993) and the posterior fossa (Courchesne et al., 1994) have been detected in infantile Autism lends credence to the contention that the enlarged brain and skull areas co-evolved in *H. sapiens*.

##### Schizophrenia

Also in schizophrenia abnormal neural oscillations and synchrony has been associated with less organization in subdivision of the corpus callosum than controls (Uhlhaas and Singer, 2010a and references therein). Most studies have focused on deficits in the generation and maintenance of coherent gamma- range oscillations (Light et al., 2006; Minzenberg et al., 2010; Kirihara et al., 2012). However, Moran and Hong (2011) after reviewing EEG studies on Schizophrenia and describing some of the key functional roles exerted by gamma, low frequencies, and their cross-frequency coupling, conclude that even isolated alterations in gamma or low frequency oscillations may impact the interactions of high and low frequency bands.

Turning now to the Corpus Callosum: Leroux et al. (2015) revealed that reduced leftward functional lateralization for language in patients with schizophrenia was correlated with altered callosal integrity, reflecting decreased, and/or slower interhemispheric communication. In addition, Peters and Karlsgodt (2015) concluded that aberrant interhemispheric communication in schizophrenia is due to disrupted maturation at adolescence, with later changes likely due to disease neurotoxicity or to abnormal or excessive aging effects. In agreement with my hypothesis, neuroimaging studies showed lower callosal integrity (through FA or RD) in either the whole Corpus Callosum (Miyata et al., 2010; Knöchel et al., 2012; Freitag et al., 2013) or, more specifically, in the splenium region (Kyriakopoulos et al., 2008; Holleran et al., 2014; Balevich et al., 2015).

### White Matter- Globular Brain Pattern

In this section I will try to make clear how the development of the white matter underlies, or rather, co-evolves with the growth of our brain. More specifically, I will try to show how the processes of myelination and energy allocation (thermodynamics) of the white matter can shed light on our pursuit of what determines the shape of our brain. [The reasons why I am using the terms “shape” or “pattern” instead of “size” are well- explained in Boeckx and Benítez-Burraco (2014a) and Hublin et al. (2015). Suffice it to mention here one of their arguments: over the course of the past 30 000 years brain size declined slightly in recent *H. sapiens*, hence it strikes me as quite biased to keep focusing on brain’s size solely.]

The idea is based on a statement in Boeckx and Benítez-Burraco (2014a): “*if the brain grows differently, it wires differently.*” I’d prefer to think of this in terms of allometric evolution, and say that “differences in brain growth and wiring co-evolve.” In line with Buckner and Krienen (2013), I deem that the most telling wiring “element” can be found in the context of myelination and synaptic plasticity. As they note: “*Myelination of the cerebrum is delayed relative to other brain structures and in humans is globally protracted compared with other primates, including chimpanzees … these collective observations suggest that the expanded cortical mantle of the human brain comprises networks that widely span the cortex without consistent feedforward/feedback connectivity and, further, that these circuits mature late into development.*”

In a similar vein, Hublin et al. (2015) take the extended period of growth during ontogeny and the delayed maturation of brain structure to contribute to our brain shape and its cognitive complexity. Prolonged human development is consequently thought to be key for the globularization developmental phase, present only in *H. sapiens* (Gunz et al., 2010, 2012). Furthermore, the fact that in humans myelination of the cortical axons is slow during childhood and extends beyond late adolescence allows their brain to “wire” while interacting with an enriched physical and cultural environment, viz. while being exposed to a vast variety of stimuli.

Even though studies showing that white matter volume increased in *H. sapiens* (Schoenemann et al., 2005; Sakai et al., 2011) are of relevance, Ventura-Antunes et al.’s (2013) observations should call our attention: building on Mota and Herculano-Houzel (2012), they drew the conclusion that cortical size is not only proportional to white matter volume, but to the average caliber and longitudinal tension along the axons of the white matter. This is how they explain that cortical size in rodents and primates scales differently: while rodents’ brains wire with constant connectivity fraction, as a uniform network with the addition of isometrically longer fibers, primates’ brains scale as a small-world network, growing through the addition of nodes that are densely intra-connected locally but only sparsely interconnected globally (Changizi, 2001; Karbowski, 2003; Sporns and Kötter, 2004; Sporns and Zwi, 2004).

Crucially, my hypothesis seems to fit very well in this picture. But the way I construe the role of the Corpus Callosum has little to do with the traditional view represented by Ringo et al. (1994). These authors set forth the idea that the strategy large brains use to compensate their conduction delays in transcallosal information transfer is not to increase the inter-hemispheric processing that depends solely on the Corpus Callosum, but to increase the intra-hemispheric amount of fibers that connect local lateralized networks. Ringo et al. (1994) and Hänggi et al. (2014) made a direct correlation between this observation and brain size; however, neither this small- world strategy nor large brain size is a specific trait of *H. sapiens*. In my opinion, humans’ identifying features should be sought in the changes our neural wiring manifests. According to the hypothesis put forward in this paper, the Corpus Callosum is of great relevance, given that it displays a unique structure, which is related intrinsically to language processing coupling I described above. Let me just remind the reader that in humans the proportion of large diameter fibers in callosal regions that interconnect primary sensory areas is higher than in macaques (Aboitiz et al., 1992) and that the fiber organization of the Corpus Callosum has nothing in common with the callosal organization reported in monkeys, where the density of callosal connections varies according to body part within sensory representations (Jones et al., 1978; Killackey et al., 1983).

In order to conceive well of what connectivity means in brain terms, we should pay attention not only to the ‘wiring’ but also to the ‘re-wiring’ of the brain. With the latter I am referring to the crucial phase of synaptic pruning in development, which proves to be very pertinent to my hypothesis. Moreover, viewed through the prism of energy allocation and thermodynamics in the brain, its relevance to brain asymmetry becomes conspicuous.

Indeed both Ventura-Antunes et al. (2013) and Hublin et al. (2015) hinted at the bearing of energy in the context of brain development. Human brains appear to use their prolonged development as a strategy to counterbalance large brains’ energetic costs. The brain is extremely thermoregulated and vulnerable to energy shortages during development, as it requires circa 66% of the basal metabolic rate for functioning and maintenance by 4.2–4.4 years, when the brain approaches its adult size and synaptic densities are maximal (Holliday, 1986; Kuzawa, 1998). This exuberance of synapses is said to be needed to allow the synapse removal required for neural network refinement (Innocenti, 1995; Innocenti and Price, 2005; from an evolutionary standpoint, thermoregulation merits additional attention, considering that Neanderthals had a different endocranial heat dissipation pattern, when compared with modern humans, but a comparable amount of heat production, something that, according to Bruner (2014), could possibly be associated with the extinction of the first).

Myelination and synaptic pruning co-operate to adjust the energy consumption of the brain. Skoyles (2012) puts it boldly: “*This relative delay of myelination maturation is also consistent with small world connectivity refinement occurring particularly in the later stages of neuromaturation during adolescence, in which distant connections are pruned to create a more hub based connectivity*…. *such network refinement depends upon the synaptic pruning that “rewires” the local area neural networks formed between neighboring area neurons.”* More importantly, Skoyles also provides a link between myelination and energy efficiency of axon transmission that can be directly linked to my hypothesis “*for the passage of each spike, a 0.5 μm unmyelinated axon costs about 12-fold more in energy than when that spike is passed through a myelinated one.*” Finally, if myelination reduces energy costs for interhemispheric communication, as it follows from the myelination and fiber structure of the Corpus Callosum, synaptic pruning allows for neuron rewiring changes that refine anterior and medial cortex to establish and refine its intrahemispheric networks (Chklovskii et al., 2004; Skoyles, 2012).

These data can also be related to how the rhythms of the brain are tuned. Feinberg and Campbell (2010) found that the increase of myelination of long axonal fibers during adolescence results in long-range connectivity through reduced slow-wave activity (delta, theta) and decreased energy consumption. According to them and Uhlhaas and Singer (2010b), developmental changes correlate with the precision of rhythmic synchrony. More concretely, Zaehle and Herrmann (2011) observed a positive correlation between posterior callosal white matter density and inter- hemispheric frequency of visually evoked gamma oscillations, indicating a clear nexus between the connectome and the dynome (Boeckx and Theofanopoulou, 2014). Finally, on the grounds that Barbato and Kinouchi (2000) find a great relationship between optimal pruning and the first learning experiences, it can be deduced that the linguistic input actually pilots the brain’s development.

### Posterior Brain and Skull Enlargement in *Homo sapiens*

**FIGURE 6:**
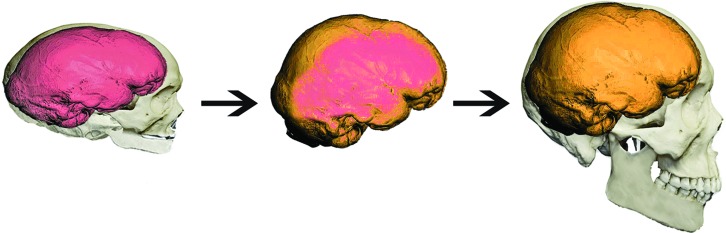
**Modern human newborns have elongated brain-skull shape**. During the ‘Globularization Phase’ (in the middle) shape changes include a relative expansion of the cerebellum and parietal bulging. Modern human adults display finally a globular cranial and endocranial shape. (Mikaella Theofanopoulou; Rights reserved).

According to Hublin et al. (2015) “*modern humans developed a more globular shape of the brain primarily resulting from a bulging in the parietal areas and a ventral flexion. In addition, modern humans display a proportionally larger cerebellum, larger olfactory bulbs and temporal lobe poles, and a wider orbitofrontal cortex*” (see **Figure 6**).

For reasons I mentioned in the Introduction, I take the posterior cortex to be of more evolutionary significance than the frontal cortex; with this I don’t mean to downgrade the acknowledged importance of the latter in higher-order language processing. It is noteworthy though that recent experiments show that the more eminent difference between humans’ and monkeys’ frontal cortex is its stronger connectivity with the sensorimotor cortex and not within frontal areas (Neubert et al., 2014). If we interpret these data in terms of co-evolution, we can say again that the anterior cortex co-evolved under the pressure of posterior cortex’s enlargement.

Turning now to the posterior cortex, my hypothesis is fostered by the findings of Bruner (2010): “*as brain size increases, the parietal lobes undergo relative flattening in non-modern humans. This pattern is stressed in Neanderthals, which show, however, a certain widening of the parietal volumes. Only H. sapiens shows a generalized enlargement of the entire parietal surface*.” Furthermore, the bulging parietals of modern humans have been linked to evolutionary reorganization of deep parietal brain areas that gave rise to the novel precuneus (see Cavanna and Trimble, 2006 for the role of precuneus in cognition).

Buckner and Krienen (2013) review an array of studies concluding that the most telling change in the evolution of our lineage (that can be connected to cortical expansion) is the enlargement of the cerebellum (expressed mostly in the dentate nucleus) and its extensive projections to association cortex. In their final assessments they note: “*The cerebellar association zones are disproportionately expanded in humans, but the functional origins and importance of cerebellar expansion remain unresolved. Adaptionist ideas… seek explanations for cerebellar enlargement as a specific, selected feature of evolution.*” Far from suggesting that the hypothesis presented here resolves the issue, I take the correlation between the myelination of the posterior Corpus Callosum and the enlargement of the cerebellum to be an insightful window into the evolution of our brain.

I side with Barton and Venditti (2014), when they propose the following: “*cerebellar specialization was a far more important component of human brain evolution than hitherto recognized and that technical intelligence was likely to have been at least as important as social intelligence in human cognitive evolution. Given the role of the cerebellum in sensory-motor control and in learning complex action sequences, cerebellar specialization is likely to have underpinned the evolution of humans’ advanced technological capacities, which in turn may have been a preadaptation for language*.”

Essentially, as brains grow during ontogeny, the bones of the skull accommodate the expanding brain. The protrusion of the posterior cranial fossa in modern humans presents a good correlation with the cerebellar lobes, and the bulging parietal bones with the parietal lobe and specifically the precuneus.

It is also remarkable that some cranial changes have been associated with the Corpus Callosum: “*In terms of evolution, shape and position of the corpus callosum are influenced by the general endocranial architecture, mainly by the flexion of the cranial base*” (Bruner et al., 2012). Furthermore, considering that the tentorium cerebelli rotates inferoposteriorly in human fetuses (Jeffery, 2002) and that the antero- posterior stretching of the Corpus Callosum length was shown to vary in humans *“due to the association between splenium and the anterior insertion of the tentorium cerebelli, caused by spatial proximity and consequent biomechanical relationships”* (Bruner et al., 2012), there can be an overlooked relationship between some cranial fossa and the Corpus Callosum.

To conclude, I have argued in this paper that the special morphology of our Corpus Callosum provides an explanatory link between the (selective) Asymmetry long thought to be the key to understanding the evolution of our specific mode of cognition and the growth pattern that results in a globular brain(case), which sets us apart from other primates. On the basis of the evidence reviewed here, it can be said that accounts that ignore the critical role and anatomical position of the Corpus Callosum fail short of capturing what makes our brain’s language-ready.

## Conflict of Interest Statement

The author declares that the research was conducted in the absence of any commercial or financial relationships that could be construed as a potential conflict of interest.
