# Corrigendum: Brain asymmetry in the white matter making and globularity

**DOI:** 10.3389/fpsyg.2015.01857

**Published:** 2015-12-14

**Authors:** Constantina Theofanopoulou

**Affiliations:** Department of General Linguistics, Universitat de BarcelonaBarcelona, Spain

**Keywords:** brain asymmetry, white matter, lateralization, brain rhythms, language

In the Acknowledgments Section of the original Hypothesis & Theory article, the last sentence erroneously reported grant number FFI2014-61888-EXP as part of funds supporting the work. The only grant was #FFI2013-43823-P, provided by Spanish Ministry of Economy and Competitiveness.

This error does not change the scientific conclusions of the article in any way.

## Conflict of interest statement

The author declares that the research was conducted in the absence of any commercial or financial relationships that could be construed as a potential conflict of interest.

